# Post-tuberculosis lung disease: a joint effort to provide better management in Europe and Brazil

**DOI:** 10.36416/1806-3756/e20250416

**Published:** 2026-03-05

**Authors:** Denise Rossato Silva, Rosella Centis, Lia D’Ambrosio, Fusun Oner Eyuboglu, Olha Konstantynovska, Giovanni Fumagalli, Luigi Codecasa, Mahfuza Rifat, Daria Podlekareva, Giovanni Battista Migliori

**Affiliations:** 1. Faculdade de Medicina, Universidade Federal do Rio Grande do Sul - UFRGS - Porto Alegre (RS) Brasil.; 2. Servizio di Epidemiologia Clinica delle Malattie Respiratorie, Istituti Clinici Scientifici Maugeri - IRCCS - Tradate, Italia.; 3. Public Health Consulting Group, Lugano, Switzerland.; 4. FOE Respiratory Clinic, Baskent University Division of Pulmonary Diseases, Ankara, Turkey.; 5. Department of Infectious Diseases and Clinical Immunology, V.N. Karazin Kharkiv National University, Kharkiv, Ukraine.; 6. Regional TB Reference Centre, Villa Marelli Institute, ASST Grande Ospedale Metropolitano Niguarda, Milano, Italia.; 7. Damien Foundation, Dhaka, Bangladesh.; 8. Centre of Excellence for Health, Immunity and Infections - CHIP - Copenhagen University Hospital, Rigshospitalet, Denmark.; 9. International Reference Laboratory of Mycobacteriology, Statens Serum Institut, Copenhagen, Denmark.; 10. Department of Respiratory Medicine and Infectious Diseases - Copenhagen University Hospital, Bispebjerg, Denmark.

Although tuberculosis can be prevented and cured, there are still 10.8 million cases of the disease and 1.25 million deaths attributed to it annually; therefore, it continues to be a clinical and public health priority worldwide.^1^ ^-^ ^4^ Because tuberculosis is more prevalent in resource-limited settings, the efforts of national tuberculosis programs have so far been focused on diagnosing the disease rapidly and treating it effectively, in order to break the chain of transmission.^1^ ^,^ ^2^ ^,^ ^5^ ^,^ ^6^ Therefore, when the patients hopefully reach the best possible outcome (cured or treatment completed) no more activities are in general undertaken. Unfortunately, an important fraction of these patients (up to 50% in drug-susceptible cases and 90% in drug-resistant cases) continue to suffer from signs and symptoms attributed to post-tuberculosis lung disease (PTLD), including cough, dyspnea, and weakness, with difficulties in performing exercise (e.g., climbing stairs), which often limit routine activities of daily living and working, as well as affecting health-related quality of life (HRQoL). Last but not least, PTLD patients have a risk of death four times higher than that of the general population.^7^ ^-^ ^16^ In global terms, it has been estimated that more than 155 million individuals survived tuberculosis and achieved treatment success in 2020,^7^ ^,^ ^8^ ^,^ ^12^ being potentially at risk of developing PTLD.

Recent evidence underlines the importance of screening patients at the moment they achieve treatment success to identify those potentially suffering from PTLD and potentially needing pulmonary rehabilitation (PR).^7^ ^,^ ^11^ ^,^ ^17^ ^-^ ^21^ There is evidence that PR is effective in this context. In a prospective multicenter study conducted in Brazil,^17^ PTLD patients underwent a comprehensive five-week PR program, and several functional parameters improved after rehabilitation, such as the FEV_1_, FVC, DL_CO_, and six-minute walk distance (6MWD), as well as the initial and final SaO_2_ during the six-minute walk test. The median post-PR increase in the 6MWD was 60 m, and almost 70% of the patients had an improvement greater than 25 m. In addition, the number of lung function parameters which were improved after PR was much higher in the patients with COPD than in those without.

Several projects have been launched on different continents to study PTLD and how to manage it at the programmatic level.^22^ ^-^ ^24^ In Europe, a research workshop (Workshop on Innovations in Tuberculosis 2025: research perspectives for Europe) was organized in May of 2025 with a focus on eastern European countries where the incidence/burden of tuberculosis and the prevalence of drug-resistant tuberculosis is still relevant. The overall aim of that workshop was to deliver high-quality programmatic innovations in tuberculosis with an emphasis on region-specific needs, in order to provide a platform for scientific interchange and education, fostering collaborations and designing research projects to achieve the 2030 targets of the World Health Organization (WHO) End TB Strategy in Europe. More specifically, the workshop had the following aims:

- To create a European dissemination platform to facilitate an unbiased scientific exchange and education on advances in tuberculosis and clinical translation of recent developments, considering regional epidemic trends and health care infrastructures
- To foster new, lasting collaborations between western and eastern European countries to strengthen tuberculosis research and management in areas with a high tuberculosis burden
- To share real-world experience and facilitate evidence-based program implementation.
- To involve early-career researchers and support their career development in tuberculosis research

In the workshop, PTLD was identified as an important topic for the development of a shared protocol involving different countries. This will be a prospective, non-controlled interventional and multicenter study to evaluate the impact of a PR program for adults with PTLD. The study will be conducted across multiple centers located in Italy, Turkey, and Ukraine. The coordinating center for this study is the WHO Collaborating Centre for Tuberculosis and Lung Diseases, in the city of Tradate, Italy, which will oversee the study implementation and data collection process with the support of Brazil, given the considerable experience that researchers in Brazil have with PTLD. The primary outcome will be the change in the 6MWD from baseline to the end of PR. The secondary outcome will be the prevalence of PTLD in different countries of the WHO European region.

In conclusion, PR is a high-impact intervention whose feasibility and acceptability have already been demonstrated in patients with PTLD.^17^ Establishing local PR protocols for PTLD is crucial for improving patient quality of life by reducing respiratory symptoms and increasing exercise capacity. Local protocols ensure programs are tailored to local resources, making them feasible and cost-effective, especially in low-resource settings. This leads to more equitable access to care, allowing better long-term management of chronic respiratory conditions resulting from PTLD and easing the burden on individuals and health care systems.
